# Association of early-onset breast cancer with body mass index, menarche, and menopause in Taiwan

**DOI:** 10.1186/s12885-022-09361-2

**Published:** 2022-03-11

**Authors:** Pei-Jing Yang, Ming-Feng Hou, Fu Ou-Yang, Eing-Mei Tsai, Tsu-Nai Wang

**Affiliations:** 1 Department of Public Health, College of Health Science, Kaohsiung Medical University, Kaohsiung, 80708 Taiwan; 2 Division of Breast Oncology and Surgery, Department of Surgery, Kaohsiung Medical University Chung-Ho Memorial Hospital, Kaohsiung, 80756 Taiwan; 3 Department of Biomedical Science and Environmental Biology, College of Life Science, Kaohsiung Medical University, Kaohsiung, 80708 Taiwan; 4 Graduate Institute of Medicine, College of Medicine, Kaohsiung Medical University, Kaohsiung, 80708 Taiwan; 5 Department of Obstetrics and Gynecology, Kaohsiung Medical University Chung-Ho Memorial Hospital, Kaohsiung, 80756 Taiwan; 6 Research Center for Environmental Medicine, Kaohsiung Medical University, Kaohsiung, 80708 Taiwan

**Keywords:** Breast cancer, Early-onset, BMI, Menarche, Menopause, Interaction, Epidemiology

## Abstract

**Background:**

The trend of women suffering from early-onset breast cancer is increasing in Taiwan. The association of early-onset breast cancer with body mass index (BMI), menarche, and menopausal status has focused interest on the field of cancer epidemiology; however, few studies have explored the interaction of these factors on early-onset risk. This study aimed to estimate the interaction effects of BMI, menarche, and menopausal status on 40-year-old early-onset breast cancer.

**Methods:**

Breast cancer patients were recruited from Kaohsiung Medical University Chung-Ho Memorial Hospital from 2013 to 2020. Multivariable logistic regression was used to estimate odds ratios (ORs) for early-onset breast cancer risk associated with menarcheal age stratified by sociodemographic factors and for the interaction between BMI and menopausal status on early-onset risk.

**Results:**

A total of 775 participants were divided into 131 early-onset cases (≤ 40 years) and 644 late-onset cases (> 40 years). Compared to the age of 13 years at menarche, the age ≤ 11 years was significantly positively associated (OR: 2.62, 95% CI: 1.38–4.97) and ≥ 16 years was negatively associated (OR: 0.13, 95% CI: 0.03–0.53) with 40-year-old early-onset breast cancer respectively. In an adjusted model, the status of BMI < 24 and premenopause had 1.76- and 4.59-fold risk of early-onset breast cancer respectively. Especially in BMI < 24 status, premenopause also had a 6.47-fold early-onset risk and the early-onset risk increased by a significant amount per one year younger at menarche (aOR: 1.26, 95% CI: 1.03–1.55). There was also a positive interaction effect on an additive scale between BMI and menopausal status on early-onset breast cancer (RERI_OR_ = 4.62, *P*_interaction_ = 0.057). Compared to both BMI ≥ 24 and peri-/postmenopausal status, both the status of BMI < 24 and premenopause were associated with early-onset breast cancer (aOR: 7.16, 95% CI: 3.87–13.25).

**Conclusions:**

This study suggests that the status of BMI < 24 and premenopause were associated with an increased risk of early-onset breast cancer and there was a positive interaction on an additive scale. Understanding how obesity and menopausal status affect early-onset breast cancer is important for drafting preventive measures for early-onset breast cancer in Taiwan.

## Background

Breast cancer is the most frequently diagnosed cancer at all ages among women, accounting for 24.5% of female cancers, and the burden of breast cancer continues to increase worldwide [1]. In most countries, data in 2018 from the International Agency for Research on Cancer (IARC) showed that age-standardized incidence rate of breast cancer at ages 60–74 years was the highest, but it has reached the highest at 45–59 years of age in Asia. In 2018, data from the Ministry of Health and Welfare (MOHW) in Taiwan showed that the median age at diagnosis of breast cancer was 56 years.

Breast cancer is a multifactorial, heterogeneous, and complex etiology disease. Adiposity, often represented by body mass index (BMI), is an important factor for breast cancer and appears to have opposite effects before and after menopause [2]. Multiple studies have found that higher BMI was positively associated with postmenopausal breast cancer risk [3–5]; however, an inverse association between obesity and breast cancer risk for premenopausal women has been reported [6]. In a large-pooled multicenter study on 758,592 premenopausal women from 19 prospective cohorts, this study also suggested that adiposity was negatively associated with breast cancer [7]. Among genetically susceptible white postmenopausal women, having a healthy lifestyle seem to be associated with decreasing breast cancer risk [8].

The interval between menarche and menopause is generally considered as the female reproductive period, because menarche and menopause refer to the onset and cessation of menstruation separately [9]. In many populations, women tend to have earlier menarcheal age [10–12]; even in Taiwan, the trend of a decline in the age of puberty continues [13]. One study found that the risk of being 45 years old with early-onset breast cancer was higher in women with menarche at 12 years of age compared to women with menarcheal age ≥ 15 years [14]. So far, few studies have explored the association between early-onset breast cancer, BMI, menarche, menopausal status, and other risk factors and the interaction between BMI and menopausal status on early-onset breast cancer.

This study aimed to estimate the association of 40-year-old early-onset breast cancer with BMI, menarche, menopausal status, and sociodemographic risk factors and the interaction effect between BMI and menopausal status on the risk of early-onset breast cancer in Taiwan and whether this could be used to improve early-onset breast cancer risk by some predictive measures during adolescence.

## Methods

### Study population

**Fig. 1:**
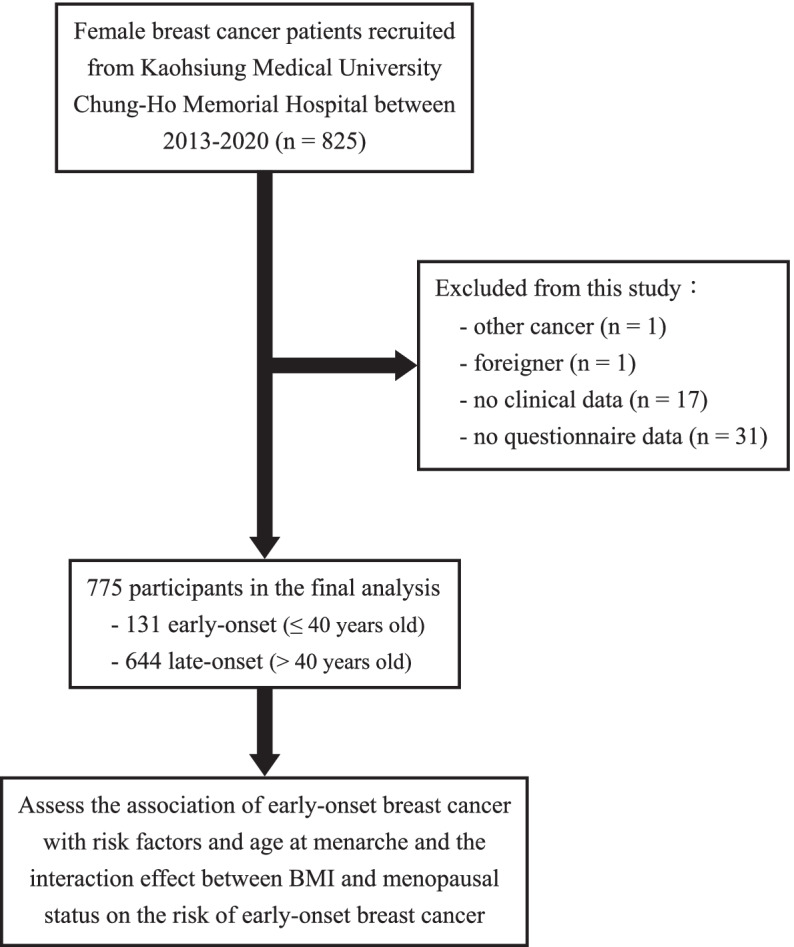
The flow chart for recruiting female breast cancer participants

From September 2013 to January 2020, this cross-sectional study included 825 female breast cancer patients diagnosed by breast surgeon physicians from Kaohsiung Medical University (KMU) Chung-Ho Memorial Hospital, a medical center in southern Taiwan. Participants were excluded if they had suffered from any other cancers (*n* = 1), were of foreign ethnicity (*n *= 1), and had no clinical or questionnaire data (*n* = 48) (Fig. 1). Finally, 775 breast cancer patients were included in the final analysis and divided into early-onset and late-onset groups based on the age of 40 years at breast cancer diagnosis. The study protocol was approved by the Institutional Review Board (IRB) of Kaohsiung Medical University (KMU) Chung-Ho Memorial Hospital (IRB No. KMUHIRB-20120104, KMUHIRB-20140055, and KMUHIRB-G(I)-20150026). All methods were carried out in accordance with the Declaration of Helsinki. All breast cancer patients provided written informed consents before the interview and biological specimen collection.

Blood and spot morning urine samples were provided from all subjects and self-administered questionnaires were collected by the trained interviewers. The contents about questionnaires included the status of weight, height, education, marriage, the use of alcohol and tobacco, physical activity, age at menarche, menopausal status, the use of oral contraceptives, parity, reproductive age, number of births, lactation history, dietary habits, benign breast disease, the family history of breast cancer, etc. The medical records of patients were followed-up every six months to update information on TNM stage (TNM classification of malignant tumors), cell grade, tumor size, cell invasiveness, the status of hormone receptors, treatment, recurrence, metastasis, and deaths.

### Statistical analysis

All participants in this study were divided into early-onset (≤ 40-year-old) and late-onset (> 40-year-old) groups to analyze the differences of continuous and categorical variables by using independent sample *t*-test, Chi-square test, or Fisher's exact test respectively. Multivariable logistic regression was performed to estimate odds ratios (ORs) and 95% confidence intervals (CIs) for the association between the risk of 40-year-old early-onset breast cancer and sociodemographic risk factors as well as women’s age at menarche and to assess the interaction effect of BMI and menopausal status on early-onset risk. According to the standard classification of the MOHW in Taiwan, BMI was categorized as non-obesity (BMI < 24 kg/m^2^) and overweight or obesity (BMI ≥ 24 kg/m^2^). The measurement for interaction effect as additive scale was relative excess risk due to interaction (RERI_OR_ = e^β1+β2+β3^—e^β1^—e^β2^ + 1) [15].

All models were also stratified by the sociodemographic characteristics to assess the risk of 40-year-old early-onset breast cancer in relation to being per one year younger at menarche in women. The adjusted ORs were estimated by adjusting the risk factors that were significantly associated with early-onset breast cancer, including BMI, education, marriage, physical activity, menopausal status, and parity. Because the associations between early-onset breast cancer and twenty-five sociodemographic and clinical characteristics were evaluated at the same time, we used Bonferroni correction (*p*-value multiplied by twenty-five risk factors) to reduce potential problems (Type I error, also known as a false positive) raised from multiple comparisons. We also performed G*Power to compute statistical power analyses for the multivariable logistic regression [16]. SPSS 22.0 and SAS 9.3 were used to perform all analyses. *P*-values less than 0.05 were statistically significant.

## Results

In Table 1, breast cancer patients are divided into 131 early-onset breast cancer patients (≤ 40 years of age, mean age at diagnosis, 35.87 years) and 644 late-onset breast cancer cases (> 40 years of age, mean age at diagnosis, 54.24 years). Compared with late-onset patients, early-onset patients had more BMI < 24 (76.3%), higher education level (67.2%), were less married (62.6%), more never exercised (51.1%), more premenopausal status (60.3%), more nulliparous (39.7%), just only one child (26.6%), delaying pregnancy beyond the age of 27 years (70.9%), more breastfeeding (73.4%), and preferred to eat fried foods (13.7%). Both early-onset and late-onset groups reflected less than 5.5% in smoking and drinking. After performing Bonferroni correction, most sociodemographic characteristics were still significant, except for physical activity and lactation history. The clinical characteristics of breast cancer are shown in Table 2. Early-onset patients had more extreme breast density (25.4%) compared with late-onset breast cancer patients.

**Fig. 2:**
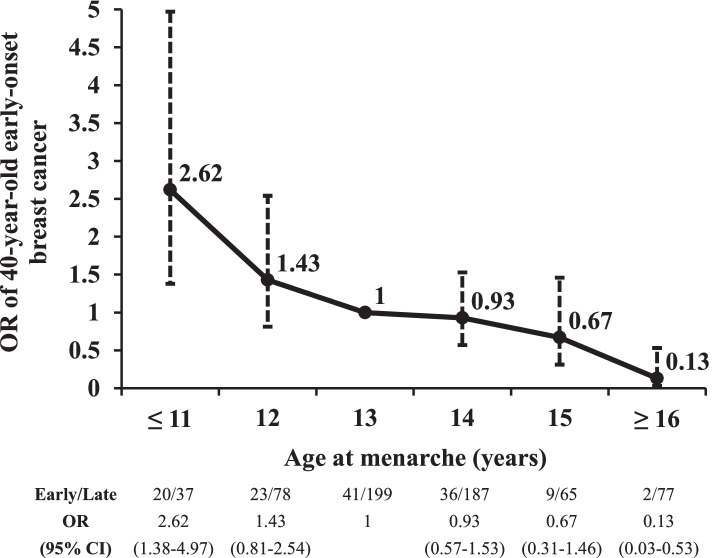
The risk of 40-years-old early-onset breast cancer by per age at menarche. The black dots represent the odds ratios (ORs) for the risk of 40-years-old early-onset breast cancer by per age at menarche and the vertical dotted lines represent 95% confidence intervals (CIs)

Compared to the age of 13 years at menarche, the age ≤ 11 years was significantly associated with early-onset breast cancer at the age of 40 years (OR = 2.62, 95% CI: 1.38–4.97, *p*-value = 0.003) and the age ≥ 16 years was significantly associated with decreased risk of 40-year-old early-onset breast cancer (OR = 0.13, 95% CI: 0.03–0.53, *p*-value = 0.005). As the age at menarche increased, the risk of early-onset breast cancer decreased (Fig. 2).

The adjusted models in Table 3, the status of BMI < 24, higher education level, premenopause, nulliparity, and breastfeeding were associated with increased risks of 40-year-old early-onset breast cancer, with aORs ranging from 1.76 to 4.59. Compared to BMI ≥ 24 status, BMI < 24 status was positively associated with early-onset breast cancer (aOR = 1.76, 95% CI: 1.09–2.84, *p*-value = 0.020); especially in the status of BMI < 24, the risk of early-onset breast cancer significantly increased by being per one year younger at menarche (aOR: 1.26, 95% CI: 1.03–1.55, *p*-value = 0.028). In the stratification of the sociodemographic characteristics, including the status of BMI < 24, higher education level, peri-/postmenopause, parous, and breastfeeding, by being per one year younger at menarche, the aORs of 40-year-old early-onset breast cancer were 1.26 to 1.38 with statistical significance. Premenopausal status was associated with 40-year-old early-onset breast cancer (aOR = 4.59, 95% CI: 2.97–7.16, *p*-value < 0.001) compared with peri-/postmenopausal status; especially in the status of BMI < 24, the association between premenopausal status and early-onset breast cancer was also significantly positive (aOR = 6.47, 95% CI: 3.76–11.13, *p*-value < 0.001).

**Fig. 3:**
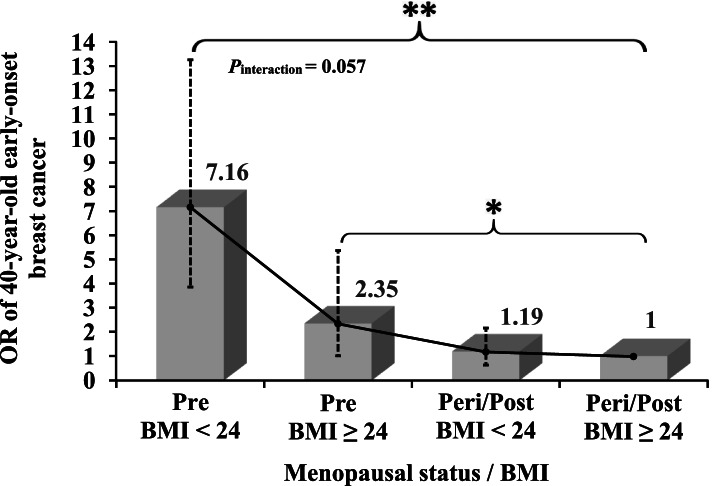
The interaction effect between BMI and menopausal status on 40-year-old early-onset breast cancer risk. Odds ratios (ORs) were calculated by multivariable logistic regression and adjusted for education, marriage, physical activity, and parity. The vertical dotted lines represent 95% confidence intervals (CIs). *P-values < 0.05, **P-values < 0.01

There was also a positive interaction effect on an additive scale between BMI and menopausal status on 40-year-old early-onset breast cancer (RERI_OR_ = 4.62, *P*_interaction_ = 0.057), shown in Fig. 3. Compared to both the status of BMI ≥ 24 and peri-/postmenopause, both BMI < 24 and premenopausal status were significantly associated with early-onset breast cancer (aOR = 7.16, 95% CI: 3.87–13.25, *p*-value < 0.001). Furthermore, in the statistical power analyses for the two important variables of BMI and menopausal status, the statistical power was 87.78% and 100% respectively.

## Discussion

In this 40-year-old early-onset breast cancer study in southern Taiwan, various potential factors were associated with early-onset breast cancer, including the status of BMI < 24, premenopause, high education level, nulliparity, and breastfeeding. There was also a positive interaction on an additive scale between BMI and menopausal status; with both the status of BMI < 24 and premenopause, the risk of 40-year-old early-onset breast cancer was more significant, and especially for BMI < 24 status, early-onset breast cancer risk increased by being per one year younger at menarche.

A meta study also analyzed the relationship between sociodemographic, reproductive factor, menarche, menopause, and breast cancer [9], and found that decreasing BMI might enhance the association between breast cancer risk and age at menarche among both premenopausal and postmenopausal women, and was especially significant in postmenopausal women, which was similar to our findings. In our study, in the status of peri-/postmenopause, BMI < 24 status significantly enhanced the association between early-onset breast cancer and age at menarche per one year younger. In our study, high education level was positively associated with early-onset breast cancer and the risk increased for being one year younger at menarche in this status. Higher education level might be associated with increasing breast cancer risk in a meta-analysis of 18 cohort studies [17] and appeared mediated by nulliparity and later menopausal age [18]. The mean age at the onset of breast cancer in Iranian women was about 10 years lower than other developed countries [19]. A hospital‐based case–control study in Iranian young women found that low parity was associated with breast cancer [20] and our study also suggested that nulliparity was related to a higher risk of early-onset breast cancer.

Because this study was about early-onset breast cancer, the premenopausal status accounted for 60.3% of early-onset cases and increased 4.59-fold risk of the early-onset breast cancer; furthermore, both the status of BMI < 24 and premenopause, the risk of early-onset breast cancer was more significant with 7.16-fold risk. Understanding how obesity affects early-onset breast cancer is also an important public health issue. Being overweight or obese in adulthood is associated with increased risks of postmenopausal breast cancer, colorectal cancer, kidney cancer, liver cancer, and pancreatic cancer. For breast cancer, obesity in adulthood is inversely associated with the risk of premenopausal breast cancer but increases the postmenopausal breast cancer risk [21]. Our study also found that both the status of BMI < 24 and premenopause were significantly associated with the increased risk of 40-year-old early-onset breast cancer. The inverse association between BMI and breast cancer risk among premenopausal women has been reported in multiple studies [6, 7, 22, 23], and a significant inverse association has been found between total estradiol concentration and BMI in premenopausal women [24–26]. The biosynthesis of estrogens differs between premenopausal and postmenopausal women. The ovary is the main source of estrogens synthesis in premenopausal women; however, after menopause, peripheral site synthesis replaces ovarian biosynthesis, and for obese postmenopausal women, peripheral aromatization of androgens in adipose tissues is the main source [27, 28]. In premenopausal women, estrogen is synthesized from androgens through the aromatase enzyme in peripheral tissues (principally subcutaneous fat) contributing about 5% of the total plasma estradiol synthesis across the menstrual cycle [7, 29]. The several mechanisms for the inverse association between BMI and breast cancer in premenopausal status are as follows. In normal weight premenopausal women, breast cells are exposed to steroidal estrogens via complex feedback-controlled [29]; however, in obese premenopausal women, higher estrogen levels produced from adipose tissues activate negative feedback in the hypothalamic-pituitary-axis leading to a decrease in circulating hormones, switching off normal ovarian function, and inducing amenorrhea while being associated with decreased risk of breast cancer [29, 30]. Obese premenopausal women may exhibit greater degree of ovulatory insufficiency and this irregular menstrual cycle might result in lower levels of estradiol and progesterone as well as lower breast cancer risk [24, 31–34].

Circulating sex hormones such as estradiol and testosterone are mainly bound to sex hormone-binding globulin (SHBG) produced by the liver. The levels of free circulating sex hormones decrease and lose bioavailability by binding SHBG [35]. Studies have found that SHBG decreased with increasing BMI [24, 25] in premenopausal and postmenopausal healthy women [26, 36]. A study of premenopausal women in the Nurses’ Health Study II found that adult BMI, even current BMI, was strongly negatively related to the levels of SHBG and total estrogen and suggested the inverse association between BMI and premenopausal breast cancer might possibly be mediated in part by sex hormones [34]. With the decline of SHBG, free estradiol should increase; however, the hypothalamic-pituitary-axis still maintains regulatory control of free estradiol levels in premenopausal women [34, 37]. In premenopausal women, estradiol concentrations and SHBG levels decreased with increasing BMI; lower estradiol levels are the result of increased estradiol clearance due to reduced serum-hormone binding capacity [6, 24, 36]. However, lower SHBG and higher estradiol were associated with obesity in postmenopausal women [38, 39], while the levels of SHBG appeared inversely related to breast cancer risk in postmenopausal women [40–42]. The above biological mechanisms might be possible explanations for the negative correlation between BMI and early-onset breast cancer in premenopausal status in our study; however, women are not encouraged to gain weight as a preventative measure against early-onset breast cancer. Further studies are required to explore the relationship between the risk of early-onset breast cancer, obesity, sex hormones, and ovarian function.

Insulin-like growth factor 1 (IGF-1) is important for the development and function of many tissues, including promoting cell proliferation and inhibiting apoptosis [43, 44]. Early-adulthood body size is associated with IGF-1, an intermediate marker for breast cancer, and overweight women have lower IGF-1 levels than lean women in youth and adolescence [45]. Many studies have suggested that IGF-1 is involved in the development of breast cancer [46–48], is associated with an increased risk of premenopausal breast cancer [49], and has poor prognosis [50] including decreased breast cancer-specific survival [51]. IGF-1 could also explain the negative correlation between BMI and early-onset breast cancer in premenopausal status of this study.

Obesity has adverse effects on health [52], although our results suggested that the risk of early-onset breast cancer with BMI < 24 status was greater than that with BMI ≥ 24 and, especially in BMI < 24 status, the risk was significantly increased by being per one year younger at menarche; however, gaining weight is not recommended as a way to decrease early-onset breast cancer risk. Our study also indicated that intake of fried food was borderline significantly associated with an increased risk of early-onset breast cancer in the adjusted model. Consumption of fried foods has also been reported to be associated with breast cancer [53], while unhealthy dietary patterns such as consumption of fried food and sugar-sweetened soft drinks appear associated with earlier menarche [54, 55]. In addition, our study found that breastfeeding increased early-onset breast cancer risk, which might explain early detection of breast cancer due to breastfeeding.

There are some limitations in our study. Firstly, this study was a cross-sectional study, so the causation of BMI status for early-onset breast cancer was difficult to clarify; however, the results of age at menarche were credible. Age at menarche occurs before breast cancer occurrence. Secondly, no environmental exposure issues were included in the questionnaire. These factors could be also related to the occurrence of breast cancer and might have interfered with the results in the study. Thirdly, Type I error was raised from multiple comparisons between early-onset breast cancer and several sociodemographic and clinical factors. Bonferroni correction was used to reduce potential problems and most sociodemographic characteristics remained statistically significant results. Furthermore, after adjusting for these confounding factors, there was a positive interaction effect on an additive scale between BMI and menopausal status on early-onset breast cancer.

## Conclusions

These findings suggested that younger age at menarche, BMI < 24 status, and premenopausal status were related to 40-year-old early-onset breast cancer and there was a positive interaction effect on an additive scale between the status of BMI < 24 and premenopause on early-onset breast cancer. Therefore, maintaining proper weight before menopause and keeping good dietary habits to prevent precocious puberty could both be beneficial to decrease the risk of early-onset breast cancer. Understanding the mechanism of the inverse association between BMI status and the risk of premenopausal early-onset breast cancer could possible modify the pathways. The results of this study require larger sample size to differentiate incident and prevalent patients with early-onset breast cancer to explore cause-and-effect relationships and perform a prospective study design to elucidate this biochemical mechanism.

**Table 1:** The sociodemographic characteristics between early-onset and late-onset breast cancer participants

**Characteristics, n (%)**	**Early-onset** **(≤ 40 years old)** **(***n*** = 131)**	**Late-onset** **(> 40 years old)** **(***n*** = 644)**	***P-*values**^**a**^	***P-*values after Bonferroni correction**
**Age at diagnosis (year)**, mean ± SD	35.87 ± 4.13	54.24 ± 8.68	< 0.001	< 0.001
**BMI (kg/m**^**2**^**)**			< 0.001	< 0.001
BMI < 24	100 (76.3)	361 (56.1)		
BMI ≥ 24	31 (23.7)	282 (43.9)		
**Education**			< 0.001	< 0.001
< college	43 (32.8)	438 (68.0)		
≥ college	88 (67.2)	206 (32.0)		
**Marriage**			< 0.001	< 0.001
Unmarried/Divorce/Widowed	49 (37.4)	125 (19.4)		
Married	82 (62.6)	519 (80.6)		
**Smoking state**			0.677	1.000
Never	124 (94.7)	615 (95.5)		
Ever	7 (5.3)	29 (4.5)		
**Alcohol intake**			1.000	1.000
Never	128 (97.7)	627 (97.4)		
Ever	3 (2.3)	17 (2.6)		
**Physical activity**			0.035	0.875
None	67 (51.1)	265 (41.1)		
Yes	64 (48.9)	379 (58.9)		
**Menopausal status**			< 0.001	< 0.001
Premenopause	79 (60.3)	108 (16.8)		
Peri-/Postmenopause	52 (39.7)	536 (83.2)		
**BMI < 24**			< 0.001	< 0.001
Premenopause	67 (67.0)	65 (18)		
Peri-/Postmenopause	33 (33.0)	296 (82)		
**BMI ≥ 24**			0.001	0.025
Premenopause	12 (38.7)	43 (15.2)		
Peri-/Postmenopause	19 (61.3)	239 (84.8)		
**Oral contraceptive use**			0.149	1.000
Never	119 (90.8)	555 (86.2)		
Ever	12 (9.2)	89 (13.8)		
**Parity**			< 0.001	< 0.001
Nulliparous	52 (39.7)	79 (12.3)		
Parous	79 (60.3)	565 (87.7)		
**Number of births**			0.001	0.025
One	21 (26.6)	72 (12.7)		
More than one	58 (73.4)	493 (87.3)		
**Age at first birth**			< 0.001	0.001
< 27 years	23 (29.1)	303 (53.7)		
≥ 27 years	56 (70.9)	261 (46.3)		
**Lactation history**			0.003	0.075
Never	21 (26.6)	251 (44.4)		
Ever	58 (73.4)	314 (55.6)		
**Fried food**			< 0.001	< 0.001
1 ~ 2 times a week	113 (86.3)	619 (96.1)		
3 ~ 6 times a week	18 (13.7)	25 (3.9)		
**Benign breast disease**			0.559	1.000
Never	98 (74.8)	497 (77.2)		
Ever	33 (25.2)	147 (22.8)		
**Family history of BC**			0.073	1.000
None	96 (73.3)	517 (80.3)		
Yes	35 (26.7)	127 (19.7)		

*SD* Standard deviation, *BMI* Body mass index

^a^*P-*values were calculated for continuous variables by Independent sample t-test and for categorical variables by Chi-square test or Fisher's exact test

**Table 2:** The clinical characteristics between early-onset and late-onset breast cancer participants

**Characteristics, n (%)**	**Early-onset** **(≤ 40 years old)** **(***n = 131)*	**Late-onset** **(> 40 years old)** (*n = 644)*	***P-*values**^**a**^	***P-*values after Bonferroni correction**
**Breast density**			< 0.001	0.0048
Fatty + Mildly dense	10 (7.9)	99 (16.5)		
Moderately	84 (66.7)	426 (71.0)		
Extremely	32 (25.4)	75 (12.5)		
**Molecular subtype**			0.322	1.000
Luminal A	78 (65.5)	379 (62.0)		
Luminal B	22 (18.5)	107 (17.5)		
HER2-enriched	7 (5.9)	71 (11.6)		
Triple negative	12 (10.1)	54 (8.8)		
**TNM stage**			0.822	1.000
0/I/II	108 (84.4)	529 (83.6)		
III/IV	20 (15.6)	104 (16.4)		
**Grade**			0.761	1.000
Well differentiated	16 (13.7)	85 (13.8)		
Moderately differentiated	65 (55.6)	321 (52.1)		
Poorly differentiated	36 (30.8)	210 (34.1)		
**Tumor size**			0.251	1.000
≤ 2 cm	68 (54.4)	371 (59.9)		
> 2 cm	57 (45.6)	248 (40.1)		
**Invasive**			0.055	1.000
None	9 (8.9)	91 (16.4)		
Yes	92 (91.1)	465 (83.6)		
**ER status**			0.272	1.000
ER-negative	22 (17.5)	138 (21.8)		
ER-positive	104 (82.5)	494 (78.2)		
**PR status**			0.270	1.000
PR-negative	37 (29.8)	220 (35.0)		
PR-positive	87 (70.2)	409 (65.0)		
**HER2 status**			0.292	1.000
HER2-negative	90 (75.6)	433 (70.9)		
HER2-positive	29 (24.4)	178 (29.1)		

*TNM stage* TNM classification of malignant tumors, *ER* Estrogen receptor, *PR* Progesterone receptor, *HER2* Human epidermal growth factor receptor 2. ^a^*P-*values were calculated for categorical variables by Chi-square test

**Table 3:** The association of 40-year-old early-onset breast cancer with sociodemographic characteristics and correlated with menarcheal age

	**The risk of 40-year-old early-onset breast cancer**		**The risk of early-onset breast cancer by per 1 year younger at menarche**		**The risk of 40-year-old early-onset breast cancer**		**The risk of early-onset breast cancer by per 1 year younger at menarche**	
**Characteristics**	**cOR (95% CI)**^**a**^	***P-*values**	**cOR (95% CI)**^**a**^	***P-*values**	**aOR (95% CI)**^**b**^	***P-*values**	**aOR (95% CI)**^**b**^	***P-*values**
**BMI (kg/m**^**2**^**)**								
BMI < 24	2.52 (1.64–3.88)	< 0.001	1.49 (1.24–1.78)	< 0.001	1.76 (1.09–2.84)	0.020	1.26 (1.03–1.55)	0.028
BMI ≥ 24	1		1.41 (1.09–1.83)	0.008	1		1.23 (0.92–1.63)	0.162
**Education**								
< college	1		1.20 (0.96–1.50)	0.110	1		1.15 (0.89–1.47)	0.282
≥ college	4.35 (2.92–6.50)	< 0.001	1.36 (1.12–1.65)	0.002	2.54 (1.62–3.98)	< 0.001	1.30 (1.04–1.62)	0.022
**Marriage**								
Unmarried/Divorce/Widowed	2.48 (1.66–3.72)	< 0.001	1.28 (0.98–1.67)	0.066	0.88 (0.44–1.77)	0.726	1.05 (0.78–1.40)	0.749
Married	1		1.49 (1.25–1.77)	< 0.001	1		1.32 (1.08–1.61)	0.008
**Physical activity**								
None	1.50 (1.03–2.18)	0.036	1.45 (1.18–1.78)	< 0.001	1.40 (0.90–2.17)	0.138	1.27 (0.99–1.62)	0.058
Yes	1		1.37 (1.13–1.67)	0.001	1		1.20 (0.96–1.51)	0.113
**Menopausal status**								
Premenopause	7.54 (5.02–11.32)	< 0.001	1.09 (0.87–1.37)	0.436	4.59 (2.94–7.16)	< 0.001	1.12 (0.86–1.46)	0.397
Peri-/Postmenopause	1		1.39 (1.13–1.71)	0.002	1		1.35 (1.09–1.69)	0.007
**BMI < 24**								
Premenopause	9.25 (5.63–15.18)	< 0.001	1.20 (0.91–1.59)	0.205	6.47 (3.76–11.13)	< 0.001	1.12 (0.83–1.51)	0.446
Peri-/Postmenopause	1		1.45 (1.09–1.92)	0.010	1		1.42 (1.05–1.91)	0.022
**BMI ≥ 24**								
Premenopause	3.51 (1.59–7.75)	0.002	1.22 (0.74–2.02)	0.435	2.18 (0.93–5.12)	0.073	1.28 (0.67–2.42)	0.457
Peri-/Postmenopause	1		1.34 (0.98–1.84)	0.067	1		1.27 (0.91–1.78)	0.161
**Parity**								
Nulliparous	4.71 (3.09–7.18)	< 0.001	1.18 (0.91–1.53)	0.220	3.10 (1.53–6.29)	0.002	1.01 (0.75–1.36)	0.964
Parous	1		1.52 (1.27–1.81)	< 0.001	1		1.33 (1.09–1.62)	0.005
**Number of births**								
One	2.48 (1.42–4.33)	0.001	1.73 (1.14–2.63)	0.011	1.60 (0.85–3.01)	0.149	1.59 (0.99–2.53)	0.054
More than one	1		1.46 (1.20–1.78)	< 0.001	1		1.28 (1.02–1.60)	0.033
**Age at first birth**								
< 27 years	1		1.33 (0.99–1.79)	0.060	1		1.19 (0.85–1.67)	0.318
≥ 27 years	2.83 (1.69–4.72)	< 0.001	1.52 (1.21–1.91)	< 0.001	1.28 (0.70–2.33)	0.421	1.35 (1.04–1.73)	0.022
**Lactation history**								
Never	1		1.29 (0.91–1.83)	0.148	1		1.21 (0.83–1.76)	0.333
Ever	2.21 (1.31–3.74)	0.003	1.62 (1.32–1.99)	< 0.001	1.87 (1.06–3.28)	0.030	1.38 (1.09–1.76)	0.008
**Fried food**								
1 ~ 2 times a week	1		1.32 (1.14–1.53)	< 0.001	1		1.19 (1.00–1.41)	0.055
3 ~ 6 times a week	3.94 (2.08–7.47)	< 0.001	1.72 (1.10–2.70)	0.019	1.97 (0.91–4.26)	0.085	1.20 (0.47–3.04)	0.707
**Benign breast disease**								
Never	1		1.37 (1.17–1.61)	< 0.001	1		1.17 (0.96–1.42)	0.117
Ever	1.14 (0.74–1.76)	0.559	1.53 (1.16–2.03)	0.003	1.11 (0.68–1.83)	0.670	1.44 (1.03–2.00)	0.031
**Family history of BC**								
None	1		1.36 (1.16–1.60)	< 0.001	1		1.20 (0.99–1.46)	0.062
Yes	1.48 (0.96–2.29)	0.074	1.56 (1.15–2.11)	0.004	1.43 (0.87–2.36)	0.163	1.32 (0.92–1.91)	0.133

*BMI* body mass index, *OR* odds ratio, *CI* confidence interval

^a^Crude odds ratio (cOR) was calculated by multivariable logistic regression

^b^Adjusted for BMI, education, marriage, physical activity, menopausal status, and parity, shown as aOR (95% CI)

## Data Availability

All data generated or analysed during this study are included in this published article.

## Abbreviations

IARC: International Agency for Research on Cancer
MOHW: Ministry of Health and Welfare
BMI: Body mass index
KMU: Kaohsiung Medical University
IRB: Institutional Review Board
TNM stage: TNM classification of malignant tumors
ORs: Odds ratios
CIs: Confidence intervals
RERI: Relative excess risk due to interaction
SHBG: Sex hormone-binding globulin
IGF-1: Insulin-like growth factor 1
